# Antithrombotic Drug Eruptions in Dermatology Practice: Selection Bias, Clinical Phenotypes, and Diagnostic Approaches

**DOI:** 10.7759/cureus.112751

**Published:** 2026-07-15

**Authors:** Tomoaki Takada

**Affiliations:** 1 Dermatology, Sumikawa Takada Dermatology Clinic, Sapporo, JPN

**Keywords:** antithrombotic agents, dermatology, direct oral anticoagulants, drug eruption, hypersensitivity reactions

## Abstract

Antithrombotic agents are among the most frequently prescribed medications in elderly patients and are essential for the prevention and treatment of cardiovascular and cerebrovascular diseases. Although cutaneous adverse reactions associated with anticoagulants and antiplatelet agents are generally considered uncommon, dermatologists frequently encounter these medications when evaluating patients presenting with pruritic eruptions. This apparent discrepancy may be explained by differences in patient populations. While cardiologists and neurosurgeons manage all patients receiving antithrombotic therapy, dermatologists evaluate a selected population enriched for cutaneous symptoms.

This review summarizes the spectrum of cutaneous adverse reactions associated with major antithrombotic agents, including warfarin, heparins, aspirin, ticlopidine, clopidogrel, cilostazol, prasugrel, and direct oral anticoagulants (DOACs). Reported reactions range from common hemorrhagic manifestations to delayed-type hypersensitivity reactions; severe cutaneous adverse reactions, such as drug reaction with eosinophilia and systemic symptoms (DRESS/DIHS), Stevens-Johnson syndrome (SJS), toxic epidermal necrolysis (TEN), vasculitis, and skin necrosis. Particular emphasis is placed on drug-specific dermatologic phenotypes and diagnostic approaches, including drug-induced lymphocyte stimulation testing, patch testing, and clinicopathological correlation.

In addition, Japanese case reports published between 1980 and 2024 are reviewed and integrated with the international literature. These data suggest that antithrombotic agents should not be regarded as a homogeneous dermatologic risk category. Instead, each drug class exhibits characteristic cutaneous reaction patterns that may assist in the differential diagnosis of these reactions in clinical practice.

Recognition of these drug-specific phenotypes may facilitate earlier diagnosis, improve interdisciplinary communication, and optimize the management of suspected antithrombotic drug eruptions.

## Introduction and background

Antithrombotic agents, including anticoagulants and antiplatelet drugs, are indispensable in contemporary cardiovascular and cerebrovascular medicine. Their use has expanded with population aging and the increasing prevalence of atrial fibrillation, ischemic heart disease, peripheral arterial disease, venous thromboembolism, and cerebrovascular disease. From a population-level safety perspective, dermatologic adverse reactions to antithrombotic agents are usually considered uncommon, and bleeding remains the best-recognized complication of anticoagulant therapy [1-4]. Classical reviews have emphasized hemorrhage, heparin-induced thrombocytopenia, osteoporosis, and coumarin skin necrosis as key adverse events [1,5-7].

In dermatology clinics, however, antithrombotic agents are encountered with surprising regularity during the evaluation of pruritic eruptions. This does not necessarily mean that these medications are frequent causes of drug eruptions. Rather, it reflects a difference in the population under observation. Cardiologists, neurologists, and neurosurgeons follow all patients who receive antithrombotic therapy, most of whom never develop cutaneous symptoms. Dermatologists see a selected subset of patients who already have pruritus, erythema, eczema, urticaria, purpura, or suspected drug eruptions. In this selected setting, a low incidence in the exposed population cannot automatically exclude a drug as a possible culprit in an individual patient [8-11].

This review aims to articulate this dermatologic perspective and to provide a practical classification of antithrombotic agent-associated cutaneous adverse reactions. It also reviews Japanese case-report patterns extracted from a national drug-eruption compendium published in Japan. The goal is not to rank the true incidence of drug eruptions, but to map drug-specific dermatologic phenotypes that may assist clinical reasoning and interdisciplinary communication.

## Review

Methods

This narrative review was developed from four complementary sources: published literature on cutaneous adverse reactions to antithrombotic therapy, prescribing and post-marketing safety information, diagnostic literature on drug hypersensitivity, and Japanese case-report entries summarized in the 21st edition of Yakushinjoho (Collected Report of Drug Eruptions in Japan), covering 1980-2024. To integrate the international literature, a comprehensive literature search was conducted in the PubMed database. The specific search terms, categorized by drug classes and dermatologic phenotypes, are summarized below.

Drug-related terms (OR): "antithrombotic", "anticoagulant", "antiplatelet", "warfarin", "heparin", "low-molecular-weight heparin", "aspirin", "thienopyridine", "ticlopidine", "clopidogrel", "cilostazol", "prasugrel", "direct oral anticoagulant", "DOAC", "apixaban", "rivaroxaban", "edoxaban".

Cutaneous outcome-related terms (OR): "drug eruption", "cutaneous adverse reaction", "hypersensitivity", "purpura", "maculopapular rash", "skin necrosis", "calciphylaxis", "DRESS", "DIHS", "Stevens-Johnson syndrome", "toxic epidermal necrolysis".

The final search query crossed the drug-related terms with the cutaneous outcome-related terms using the Boolean operator "AND". No strict date restrictions were applied to capture historical phenotypes, such as ticlopidine-induced reactions.

Selection bias of dermatology clinics

**Figure 1:**
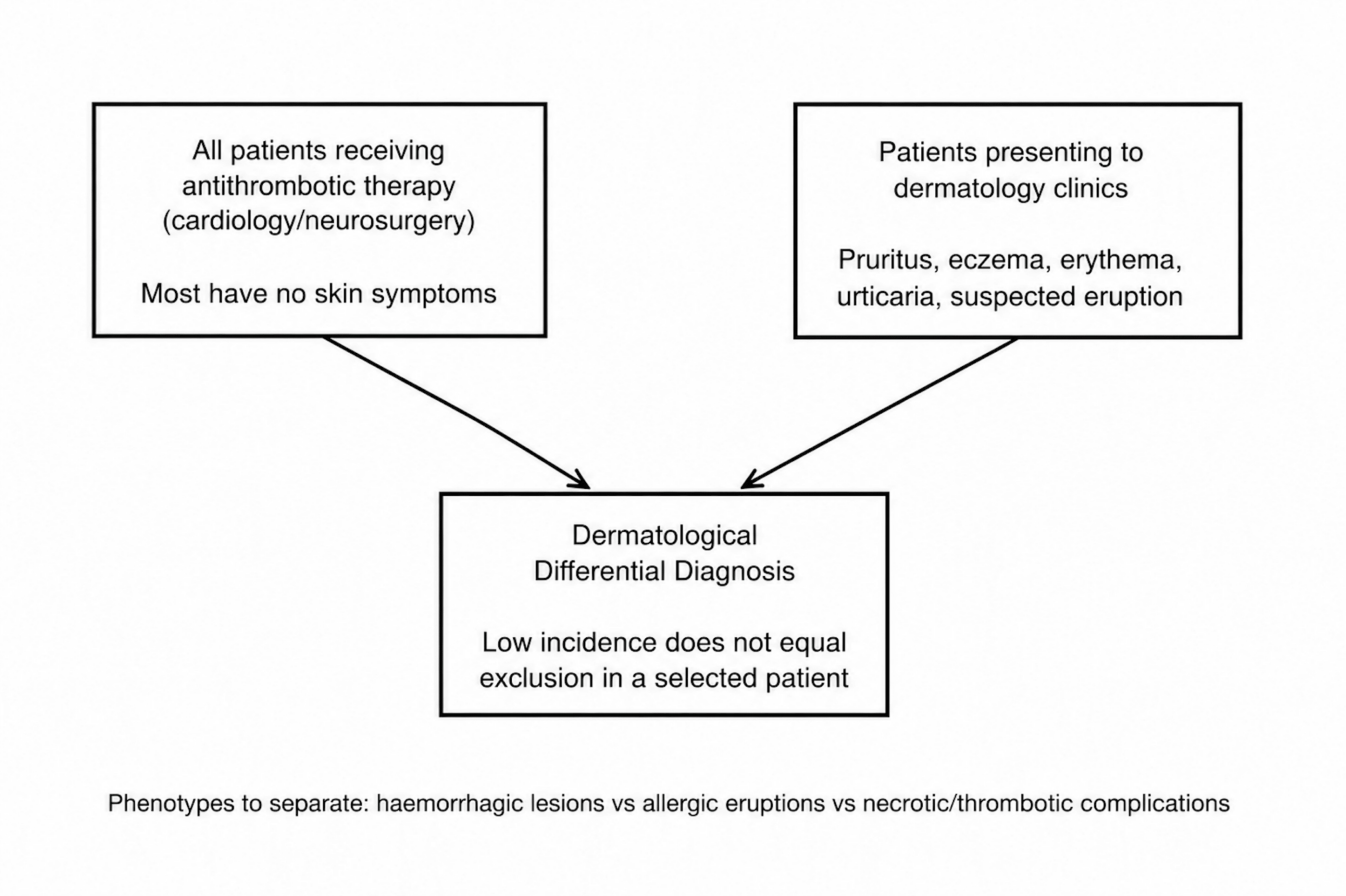
Selection Bias Underlying Dermatologic Evaluation of Antithrombotic Drug Eruptions Cardiologists and neurosurgeons evaluate the entire population of patients receiving antithrombotic therapy, most of whom never develop cutaneous symptoms. In contrast, dermatologists evaluate a selected population presenting with pruritus, eczema, erythema, urticaria, or suspected drug eruptions. Consequently, even medications associated with a low incidence of cutaneous adverse reactions may remain important differential diagnostic considerations in dermatology practice.

The central clinical issue is selection bias. A patient receiving antithrombotic therapy in a cardiology clinic is one member of a large exposed population. A patient receiving antithrombotic therapy who presents to a dermatology clinic with pruritus or eruption is already enriched for cutaneous disease. Thus, the probability that an antithrombotic agent is relevant is not the same in the two settings. The dermatologist's diagnostic task is not to estimate the drug's absolute population incidence, but to decide whether the temporal course and morphology of the eruption make the drug plausible in the individual patient (Figure 1).

This distinction is particularly important because many antithrombotic agents are essential for the prevention of stent thrombosis, embolic stroke, recurrent venous thromboembolism, and other high-risk events. Dermatologists rarely request substitution or interruption lightly. Such a request usually reflects an attempt to balance dermatologic causality assessment with thrombotic risk, rather than a claim that the antithrombotic agent is definitively responsible [12,13].

Classification of cutaneous adverse reactions

Cutaneous findings associated with antithrombotic therapy can be separated into at least four categories. First, hemorrhagic cutaneous manifestations include purpura, petechiae, ecchymoses, subcutaneous hemorrhage, and hematoma formation. These are pharmacologic consequences of impaired hemostasis and should not be classified as allergic drug eruptions. Their inclusion under the broad organ-system category of skin and subcutaneous tissue disorders may overestimate the burden of true allergic cutaneous reactions [2-4].

Second, delayed-type hypersensitivity reactions represent the main category of true allergic cutaneous drug reactions. These may manifest as morbilliform eruptions, eczematous dermatitis, erythematous plaques, pruritic eruptions, and generalized exanthema. Reactions to heparins are particularly well documented and often appear as infiltrated erythematous plaques or indurated lesions at injection sites, usually after a sensitization period [6,7,10,11].

Third, severe cutaneous adverse reactions include drug reaction with eosinophilia and systemic symptoms/drug-induced hypersensitivity syndrome (DRESS/DIHS), Stevens-Johnson syndrome (SJS), toxic epidermal necrolysis (TEN), and acute generalized exanthematous pustulosis. Although these events are rare, their potential severity justifies attention when a compatible clinical picture is present [14-19].

Fourth, special thrombotic or necrotic skin disorders must be recognized. Warfarin-induced skin necrosis is the prototype. It is not a classical allergic eruption but a thrombotic complication related to transient hypercoagulability, often discussed in relation to the early decline of protein C or protein S [20-23]. Heparin-associated skin necrosis may also occur, particularly in the context of heparin-induced thrombocytopenia, and may initially resemble a localized inflammatory plaque [24-26].

Drug-specific dermatologic phenotypes

Among antiplatelet agents, thienopyridines present a highly contrasting dermatological footprint. Ticlopidine represents the historical peak of antiplatelet-induced cutaneous adverse events in Japan, with 28 entries covering an exceptionally broad spectrum, including lichenoid eruptions, eczema, erythroderma, pemphigus-like eruptions, fixed drug eruptions, and fatal TEN [27-30]. Although its clinical use has sharply declined due to systemic toxicities such as thrombotic thrombocytopenic purpura, its extensive historical data provide critical insights into antiplatelet-induced skin disease. In contrast, its modern successor, clopidogrel, showed fewer entries (eight cases) in the Japanese compendium but was associated with clinically profound, life-threatening severe reactions, including SJS and DRESS/DIHS [31,32]. This underscores the clinical consensus that clopidogrel hypersensitivity, while less frequent, most commonly presents as a severe delayed eruption. When thrombotic risk is exceptionally high, such as immediately following coronary stent placement, carefully coordinated dermatological management with oral steroids may allow continuation of therapy without interruption [33-39]. Severe reactions and complex cases under modern multi-drug regimens have also been documented, highlighting the clinical complexity [40]. Cross-reactivity between clopidogrel and ticlopidine remains an important consideration [30].

Aspirin and aspirin-containing products accounted for 12 entries, presenting a clinical spectrum of purpura, urticaria, granulomatous eruptions, and fixed urticaria, alongside rare severe cutaneous reactions such as SJS/TEN and DIHS/DRESS [41-43]. It is critical to note that aspirin-induced hypersensitivity often belongs to a broad, non-immunological cyclooxygenase-1 inhibition spectrum rather than classical T-cell-mediated exanthema, occasionally necessitating rapid desensitization protocols in acute coronary syndromes [42]. Cilostazol appeared infrequently, with five entries, primarily associated with eczematous dermatitis and photosensitivity, with limited literature establishing true independent allergic causality due to frequent multi-drug confounding [44]. Warfarin entries in the Japanese data were strictly limited to 15 cases, heavily dominated by non-allergic, vascular-occlusive pathologies such as purpura, calciphylaxis, and skin necrosis [20-23,45]. This confirms the clinical perspective that warfarin's primary dermatologic risk profile is thrombotic or necrotic rather than a classical T-cell-mediated allergic exanthem. In daily practice, clinicians must distinguish these localized microvascular thrombotic events from typical maculopapular drug eruptions, as their underlying pathophysiology involves transient hypercoagulability rather than immune-mediated hypersensitivity [45].

Allergic reactions to heparins represent a well-defined clinical entity, with heparin calcium accounting for 15 reported entries and heparin sodium for four entries in Japan. Heparin calcium was associated predominantly with localized delayed-type hypersensitivity reactions, manifesting as injection-site erythema, infiltrated erythematous plaques, and eosinophilic panniculitis. Conversely, heparin sodium entries featured immediate-type hypersensitivity, including urticaria and anaphylaxis, alongside necrotic lesions. Reviews of heparin-induced skin lesions emphasize that allergic delayed plaques and heparin-induced thrombocytopenia-associated necrosis may look remarkably similar at onset but require fundamentally different management [6,7,24-26,45]. Cross-reactivity among low-molecular-weight heparins is high, whereas alternative agents such as fondaparinux or danaparoid remain clinically relevant, especially during pregnancy [25,26].

Direct oral anticoagulants (DOACs) demonstrated an exceptionally low frequency of allergic cutaneous reactions in the reviewed Japanese data. Apixaban accounted for only two entries (alopecia areata and erythema multiforme), and edoxaban for a single entry of SJS, while rivaroxaban was not identified within the reviewed compendium pages. This striking paucity suggests that DOACs are dermatologically secure options in the Japanese population. Nevertheless, international literature includes delayed hypersensitivity and severe reactions to non-vitamin K oral anticoagulants, including rivaroxaban-associated cutaneous reactions and possible switching to apixaban [8,9,45-47]. Pharmacovigilance data also remind clinicians that cutaneous bleeding and allergic cutaneous reactions should be separated analytically [47]. In addition, drug-laboratory interactions during transition from factor Xa inhibitors to heparin therapy may complicate anticoagulant management and reinforce the need for close interdisciplinary coordination [48]. Prasugrel showed only two entries of milder inflammatory patterns, reflecting its clean dermatological profile or shorter market exposure, while cilostazol configurations under multi-drug regimens require careful scrutiny [36,45,49].

Diagnostic evaluation in dermatologic practice

**Figure 2:**
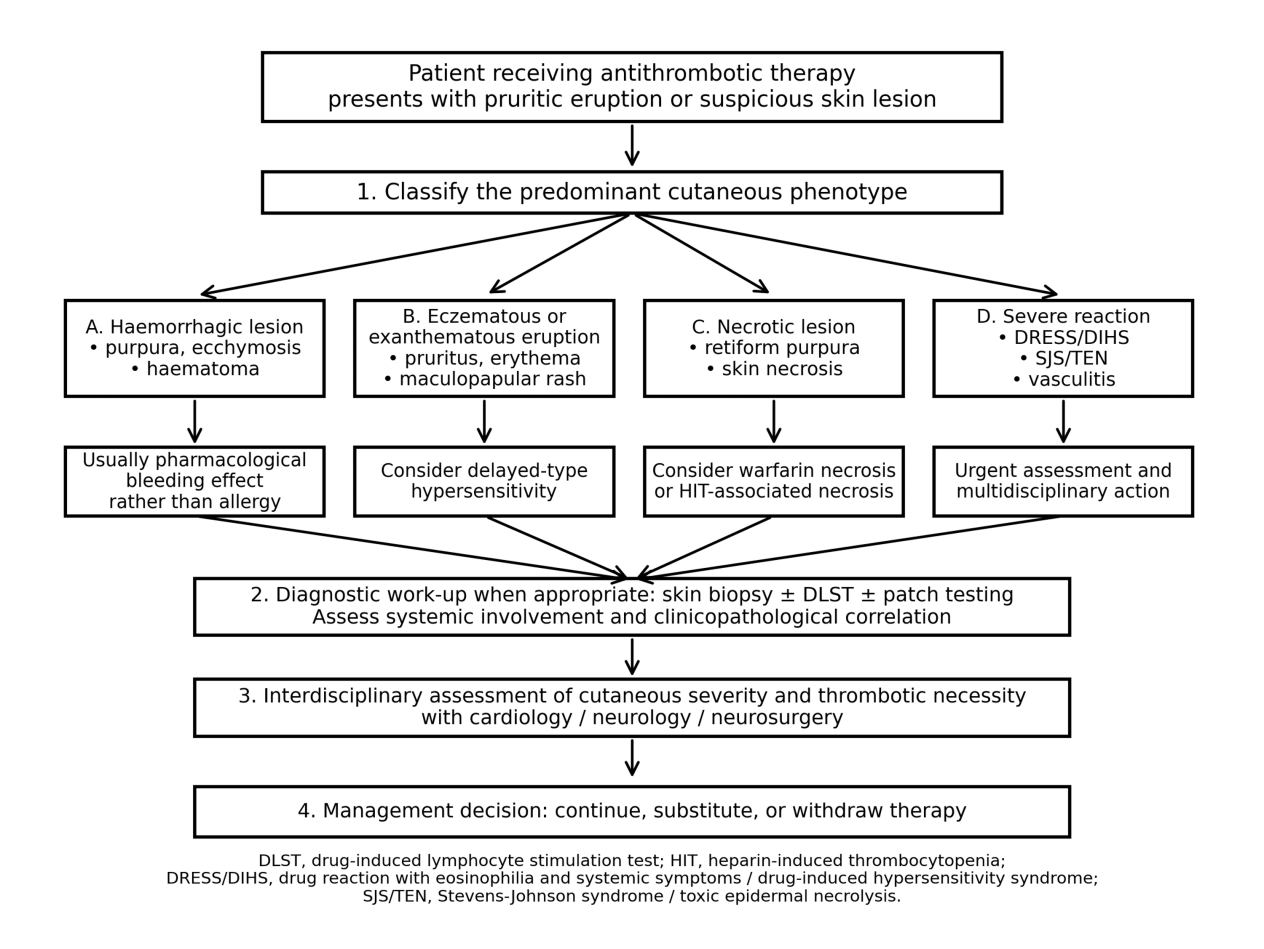
Practical Diagnostic Algorithm for Suspected Antithrombotic Drug Eruptions This figure presents a practical approach to patients receiving antithrombotic therapy who develop pruritic eruptions or suspicious skin lesions. The algorithm first separates hemorrhagic lesions, eczematous or exanthematous eruptions, necrotic lesions, and severe cutaneous adverse reactions, then integrates diagnostic work-up, clinicopathological correlation, and interdisciplinary thrombotic-risk assessment before continuing, substituting, or withdrawing therapy. Importantly, decisions regarding continuation, substitution, or withdrawal of antithrombotic therapy should be based on a balanced assessment of dermatologic severity and thrombotic risk. Dermatologists should communicate objective information regarding mucosal involvement, systemic symptoms, body surface area involvement, and histopathological findings, while the primary physician evaluates cardiovascular or cerebrovascular thrombotic risk.

Diagnostic evaluation of suspected drug eruption should begin with a complete medication history, latency period, morphology, distribution, systemic symptoms, eosinophilia, liver and renal involvement, infection status, and the clinical course after withdrawal [12]. This time-conscious assessment is especially important for SJS/TEN and DRESS/DIHS, in which early recognition and discontinuation of the culprit drug may be lifesaving [14-19]. Figure 2 summarizes a practical diagnostic and interdisciplinary management algorithm for patients receiving antithrombotic therapy who present with pruritic eruptions or suspicious skin lesions.

In vitro and in vivo testing can support, but rarely replace, clinical judgment. Drug-induced lymphocyte stimulation testing may be useful in selected delayed drug reactions, but its sensitivity, specificity, and timing vary by culprit drug and clinical phenotype [12,13]. For heparin allergy, basophil activation testing and other in vitro approaches have been proposed, but availability and validation remain limited [10,11,45]. Patch testing and delayed intradermal readings may be useful for nonimmediate reactions to heparins and some DOACs, but skin testing is contraindicated when necrosis from heparins or coumarins is suspected [6,8,9,45].

Skin biopsy remains useful when the morphology suggests vasculitis, TEN, bullous eruption, neutrophilic dermatosis, or thrombotic/necrotic disease. Histopathology may help distinguish anticoagulant-related purpura, leukocytoclastic vasculitis, heparin-induced necrosis, calciphylaxis, and classical exanthematous drug eruption [4,24,30,45].

The Japanese case-report compendium and the international literature demonstrate that antithrombotic agents are dermatologically heterogeneous, exhibiting distinct reaction profiles across different drug classes [50]. Although drug-induced lymphocyte stimulation testing remains a valuable adjunctive diagnostic tool in Japan, clinicians should recognize its potential for false-negative results. Therefore, diagnosis should always integrate temporal relationships, clinical morphology, histopathological findings, and response to drug withdrawal, rather than relying solely on laboratory testing [50].

Implications for interdisciplinary communication

When a dermatologist asks whether an antithrombotic agent can be substituted, the request may be interpreted by other specialists as an overestimation of drug causality. In reality, the dermatologist may be acknowledging uncertainty while attempting to remove a plausible but low-frequency culprit in a symptomatic, selected patient. The dermatologist's suspicion is usually based on chronology, morphology, distribution, eosinophilia, biopsy findings, skin testing, drug-induced lymphocyte stimulation testing, dechallenge, and sometimes accidental rechallenge [12,13,44-48].

This approach has implications for patient safety. Unnecessary discontinuation of antithrombotic therapy may expose patients to serious thrombotic events. Conversely, dismissing an antithrombotic agent solely because its reported drug eruption frequency is low may delay recognition of a true drug reaction, including severe cutaneous adverse reactions. A shared framework between dermatologists, cardiologists, neurologists, and neurosurgeons is therefore essential (Figure 2).

Limitations

This review has limitations. The reported entries summarized in Table 1 were not standardized by prescription volume, market share, duration of clinical availability, or patient exposure. Therefore, the table should be interpreted as a phenotypic map of reported cutaneous reactions rather than a comparative estimate of incidence or risk. The Japanese data summarized here represent reported entries in a case-report compendium, not systematically adjudicated incidence rates [50]. Reporting bias, duplicate reporting, regional publication practices, changes in prescribing patterns, and changes in diagnostic terminology over 44 years may influence the apparent distribution of reactions. In addition, drugs with longer market histories, such as ticlopidine and warfarin, may have accumulated more reports than newer agents.

**Table 1:** Reported Cutaneous Adverse Reactions to Major Antithrombotic Agents in Japan (1980-2024) † This table is original, compiled, and created by the author based on the national drug eruption compendium. Reported entries were extracted from Fukuda [50]. They represent published Japanese case reports and conference abstracts and do not reflect incidence rates. Numbers are not standardized by prescription volume, market duration, or patient exposure. Publication bias, duplicate reporting, and changes in terminology over time cannot be excluded. DOAC, direct oral anticoagulant; DIHS, drug-induced hypersensitivity syndrome; DRESS, drug reaction with eosinophilia and systemic symptoms; SJS, Stevens-Johnson syndrome; TEN, toxic epidermal necrolysis

Drug	Reported entries^†^	Representative clinical patterns	Severe reactions	Dermatological interpretation	Key references
Warfarin	15	Purpura, fixed drug eruption, calciphylaxis, skin necrosis	Warfarin-induced skin necrosis, lower-limb or toe necrosis	Thrombotic/necrotic phenotype rather than classical allergic exanthem	[1,5,6,20-23,45,50]
Heparin calcium	15	Injection-site erythema, infiltrated erythema, eosinophilic panniculitis	Skin necrosis; heparin-induced thrombocytopenia-associated lesions	Delayed-type hypersensitivity at injection sites; must distinguish from early necrosis	[5,7,10,11,24-26,45,50]
Heparin sodium	4	Urticaria, purpura, injection-site reactions	Anaphylaxis; skin necrosis	Immediate-type reactions are uncommon; necrosis suggests systemic risk	[5-7,10,11,24-26,45,50]
Apixaban	2	Alopecia areata, erythema multiforme	None in the Japanese entries reviewed	Few Japanese reports; reactions remain possible at the patient level	[8,9,45,46,50]
Edoxaban	1	Stevens-Johnson syndrome	SJS	Rare but potentially severe direct oral anticoagulant reaction	[8,9,45,50]
Rivaroxaban	Not identified in the extracted Japanese compendium pages	Rash, hypersensitivity reactions in international reports	Rare severe reactions reported in the DOAC class	Not prominent in the Japanese compendium pages reviewed; switching to another DOAC may be feasible in selected cases	[8,9,45-47,50]
Aspirin/aspirin-containing products	12	Purpura, urticaria, granulomatous drug eruption, fixed urticaria	DIHS/DRESS, SJS, TEN	Broad NSAID-hypersensitivity spectrum; desensitization may be needed when antiplatelet therapy is essential	[41-43,45,50]
Clopidogrel	8	Morbilliform eruption, erythematous eruption, toxicoderma, generalized exanthema	DIHS/DRESS, SJS	Frequently associated with delayed hypersensitivity reactions in clinical practice, severe reactions have been reported.	[30-40,45,50]
Ticlopidine	28	Lichenoid eruption, eczema, erythroderma, pemphigus-like eruption, maculopapular eruption, fixed drug eruption	TEN/Lyell syndrome	Largest number and broadest phenotype among antiplatelet agents in the Japanese compendium	[27-30,45,50]
Cilostazol	5	Eczema, photosensitivity, polymorphous erythema, urticaria	Severe reactions have been reported, although causality remains uncertain.	Limited and often confounded literature; less established culprit than thienopyridines	[44,45,49,50]
Prasugrel	2	Erythema multiforme, interstitial granulomatous drug reaction	None identified in the Japanese entries reviewed	Very limited data	[36,45,50]
Beraprost	8	Lichenoid eruption, erythema, maculopapular eruption, eczema	None identified in the Japanese entries reviewed	Predominantly chronic inflammatory dermatoses	[50]
Limaprost	3	Photosensitivity, maculopapular eruption, eczema	None identified in the Japanese entries reviewed	Limited number of reports, mainly eczematous/photosensitive reactions	[50]

It should be noted that while ticlopidine accounts for the highest number of reported cases in Table 1 due to its historical use over four decades, its clinical prescription in Japan has markedly declined in recent years because of serious adverse effects, including thrombotic thrombocytopenic purpura. Conversely, the relatively small number of reports involving DOACs likely reflects their shorter period of clinical availability rather than a lower intrinsic risk of hypersensitivity.

Despite these limitations, phenotype mapping is clinically valuable. It explains why dermatologists may continue to consider antithrombotic agents in the differential diagnosis of pruritic eruptions, even when the expected frequency is low. It also helps avoid category errors, such as confusing purpura caused by anticoagulation with allergic drug eruption or overlooking warfarin-induced skin necrosis because it does not resemble a typical exanthem.

## Conclusions

Antithrombotic agents are not generally high-risk drugs for allergic cutaneous eruptions. However, dermatologists evaluate a selected population of symptomatic patients, and in that context, low-frequency reactions remain diagnostically relevant. Antithrombotic agents have drug-specific dermatologic phenotypes rather than a uniform class effect. Recognizing the distinction between epidemiologic incidence and patient-level diagnostic suspicion may improve collaboration among dermatologists, cardiologists, neurologists, and neurosurgeons and may help prevent both unnecessary drug withdrawal and missed drug-induced skin disease.
